# Smartphone addiction in Iranian schoolchildren: a population-based study

**DOI:** 10.1038/s41598-024-73816-8

**Published:** 2024-09-27

**Authors:** Alireza Azizi, Mohammad Hassan Emamian, Hassan Hashemi, Akbar Fotouhi

**Affiliations:** 1 Student Research Committee, School of Medicine, Shahroud University of Medical Sciences, Shahroud, Iran; 2 Ophthalmic Epidemiology Research Center, Shahroud University of Medical Sciences, Shahroud, Iran; 3 Noor Ophthalmology Research Center, Noor Eye Hospital, Tehran, Iran; 4 Department of Epidemiology and Biostatistics, School of Public Health, Tehran University of Medical Sciences, Tehran, Iran

**Keywords:** Smartphone, Smartphone addiction, Students, Addiction, Iran, Epidemiology, Population screening

## Abstract

Smartphone addiction is a public health problem and is increasing globally. It is important to know the latest status of this phenomenon in different region of the world. This study aimed to investigate smartphone addiction in an Iranian student population. The participants were a random sample of Shahroud Schoolchildren Eye Cohort study in 2018. Smartphone addiction was measured by the Smartphone Addiction Scale Short Version (SAS-sv) questionnaire. The prevalence and mean estimates were reported with 95% confidence intervals. The analysis of variance, t-test and multiple logistic regression were used for analysis of data. The mean age of 2682 participating students was 13.5 year with an age range of 10 to 15 year, and 1197 (44.6%) of them were girls. The mean SAS-sv score was 27.96 (95% CI: 27.36–28.58) for boys and 26.10 (95% CI: 25.34–26.80) for girls. This score follows a U-shaped pattern with a lower mean score observed at age 8. The Prevalence of smartphone addiction was 29.8% (95% CI: 28.1–31.5) in studied population. Compared to 10 year old students, smartphone addiction was higher in 14 (Odds Ratio (OR) = 1.7, 95% CI: 1.1–2.7) and 15 (OR = 2.4, 95% CI: 1.5–3.8) years old students. Smartphone addiction was higher in boys (OR = 1.9; 95% CI, 1.5–3.0; P value < 0.001). Smartphone addiction was not different in urban and rural students after adjusting for age and gender (P = 0.089). Compared to European countries the prevalence of smartphone addiction in Iranian students is relatively high and need prompt intervention especially in boys and older adolescents.

**Supplementary Information:**

The online version contains supplementary material available at 10.1038/s41598-024-73816-8.

## Introduction

The progress and growth of technology in the 21st century has had many effects on different parts of people’s lives. One of these technologies is smart phones, which provide easy and fast access to news sites, email, applications, and social media with their many features such as the ability to connect to the Internet. The existence of various entertainments such as games, camera and playing music has made people, especially at younger ages, welcome this expanding technology. The number of smartphone users has grown dramatically^1^. The current global number of smartphone users is expected to range from 4.9^1^ to 7.1^2^ billion people in 2024 with projections indicating a further rise to 7.8 billion in 2028^2^. In 2023, a surprising 78% of individuals aged 10 and above worldwide own mobile phones^3^. There are similar patterns of increasing mobile phone usage in Iran, so that the number of smartphone users increased from 2 million users in 2014 to about 48 million in 2017^4^. France (82.6%), the United Kingdom (82.2%), and Germany (81.9%) had the highest smartphone penetration rates in 2022. Iran was ranked 7th with a penetration rate of 74.4% for smartphones^5^.

Excessive smartphone use has been associated with higher rates of road traffic accidents and deaths^6,7^, higher stress^8^, higher anxiety and depression^9–11^, poor social relationships^12^, and sleep disorders^13,14^. Low physical activity, excessive consumption of fast food and weight gain^15^, dry eyes^16^ and certain types of psychological damage^17^ are other complications of excessive use of smart phones. Excessive use of smartphones has been associated with dysfunction in daily life activities and symptoms similar to recognized addictive disorders^18^. The American Psychological Association (APA) was defined addiction as “a state of psychological and/or physical dependence on the use of drugs or other substances, such as alcohol, or on activities or behaviors, such as sex, exercise, and gambling”^19^. Some researchers believe that behaviors that have the presentation of addiction, including excessive use, impulse control problems, and negative consequences, should not be classified as addiction. They have suggested using different terminology, such as problematic use, to describe these technology-related behaviors^20^. Although gambling disorder was recognized by the APA as a behavioral addiction in Diagnostic and Statistical Manual of Mental Disorders (DSM-5), other addictive behaviors are not classified as impulse disorders^21,22^.

Problematic smartphone use is a growing concern and a public health problem worldwide^23,24^. The highest rates of problematic smartphone use were found in China and Saudi Arabia, according to a meta-analysis study that analyzed 81 studies from 24 countries. On the other hand, the lowest rates were observed in Germany and France^24^.

Considering the importance of the problematic smartphone use and the increase in the use of smart phones among teenagers, as well as the lack of information about the state of dependence of teenagers on smart phones in Iran, this study aimed to determine prevalence of the potential smartphone addiction and its associated factors in Iranian schoolchildren. It is hypothesized that a considerable portion of students have problematic use of mobile phones and the prevalence of potential smartphone addiction varies by demographic variables.

## Methods

The participants in this study were the students who participated in the second phase of the Shahroud Schoolchildren Eye Cohort Study (SSCECS). The first phase of SSCECS was started in 2015 with the participation of 5620 students aged 6 to 12 years old^25^. After 3 years, in 2018 by calling the participants in the first phase, the second phase of the study was conducted with the participation of 5292 students. Sampling methods of SSCECS have been previously reported^25^. Among the participants of the second phase of the study, a subsample of 2682 was randomly selected and this study was conducted on these people.

To measure the state of smartphone addiction, the shortened form of the smartphone addiction scale questionnaire (SAS-sv) was used. This questionnaire is a shortened form of smartphone addiction scale (SAS) which was developed by Kwon in 2013^26^. The Persian version of SAS-sv has already been prepared and its validity and reliability have been confirmed^27,28^. This questionnaire contains 10 questions which are completed by the participant based on self-report with a Likert scale of 6-points: (1: strongly disagree, 2: disagree, 3: weakly disagree, 4: weakly agree, 5: agree, 6: strongly agree) (Appendix 1). Each person can get a score between 10 and 60 points and a higher score indicates a higher risk of addiction. According to the recommendations of the creators of the questionnaire^29^, a score of ≥ 31 in males and a score of ≥ 33 in females were considered as potential smartphone addiction. Based on the above definition a dichotomous variable was computed to divide students into two groups (potentially addicted vs. non-addicted). The internal consistency reliability of the SAS-sv questionnaire was assessed by calculating McDonald’s omega. The average score of SAS-sv and the proportion of students who were potentially addicted to smartphone were reported according to age, gender and place of residence. The difference between the averages in subgroups was tested with independent t-test and analysis of variance (ANOVA). The association between age, sex, and residence place of participants as independent variables, and potential smartphone addiction, was analyzed using a multiple logistic regression model. Statistical tests were used after assessing their assumptions, and necessary corrections were made for the t-test and ANOVA, where the variances were not equal in the sex and age subgroups, respectively.

## Results

Among of the 5292 participants in the second phase of SSCECS, the SAS-sv was completed for 2682 students. The age range of the participants was between 10 and 15 years, their mean age was 13.47 ± 1.58 and 1197 (44.6%) of them were girls. The McDonald’s omega was 0.80, which indicates good reliability of the SAS-sv questionnaire.

**Fig. 1:**
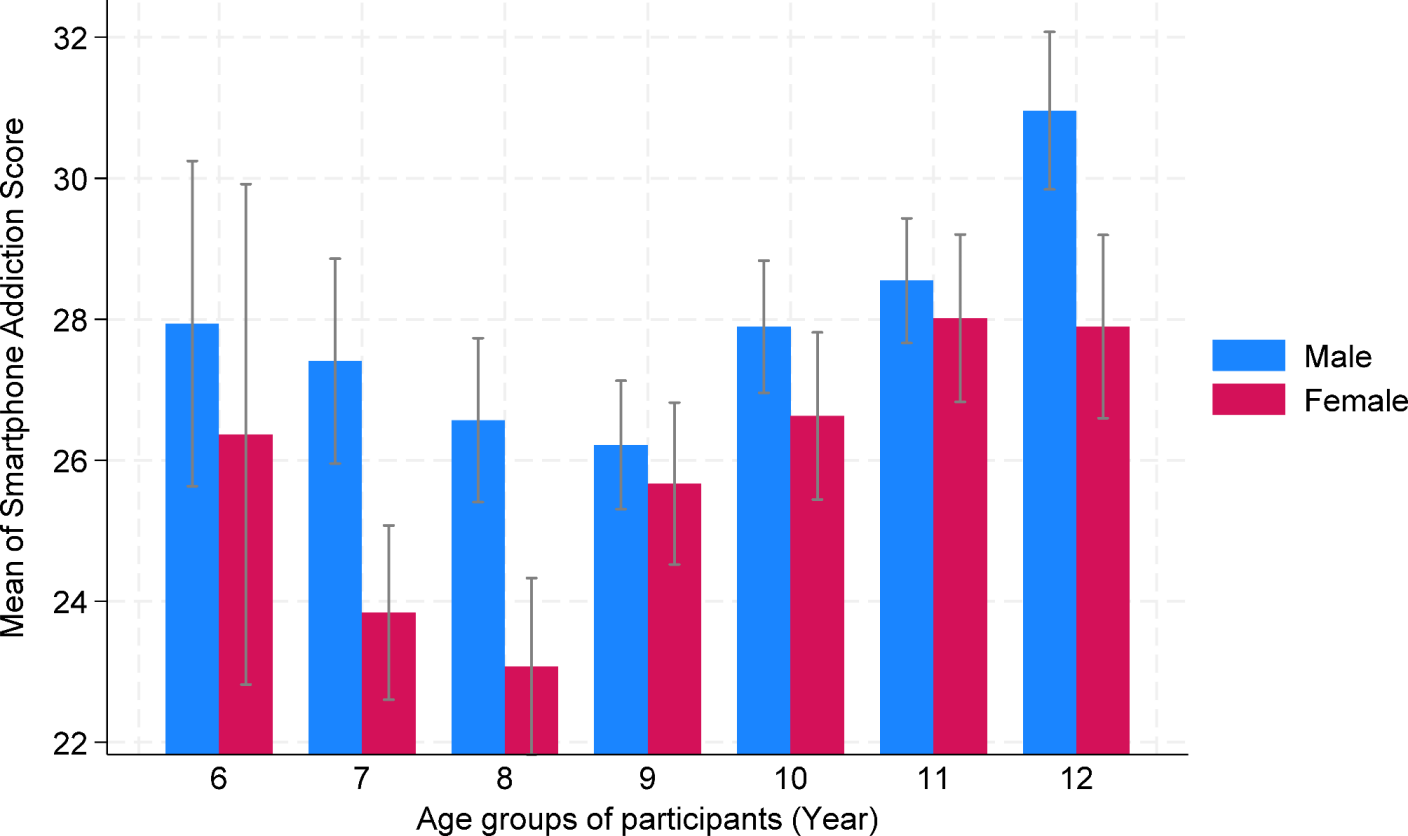
The comparison of the mean smartphone addiction score by age and sex groups. Error bars indicate 95% confidence intervals for means.

Table 1 shows the answers of participants to questions in SAS-sv questionnaire. The range of total score obtained from questionnaire was 10 to 60 and its mean was 27.12 ± 8.72. The mean score of SAS-sv according to gender, age and place of residence are shown in Table 2. The mean score of the questionnaire was higher in boys (28.02) compared to girls (26.18) (t = 5.4, df = 2490, *P* < 0.001), and older children compared to young children (F = 15.06, df= (6, 299.8), *P* < 0.001), although the effect sizes were small. The pairwise comparison of the mean smartphone addiction score by age groups is presented in Table 3. Figure 1 provides additional details on the differences in mean smartphone addiction scores across different age and sex groups. The age distribution of smartphone addiction scores follows a J-shaped pattern, with a lower mean score observed in students aged 8, particularly among girls.

**Table 1:** The number and percentage (in parentheses) of responses to the questions of Smartphone Addiction scale– short version. *N* = 2682.

Questions	Strongly disagree	Disagree	Weakly disagree	Weakly agree	Agree	Strongly agree
Missing planned work due to smartphone use	508 (18.9)	1,139 (42.5)	381 (14.2)	353 (13.2)	250 (9.3)	51 (1.9)
Having a hard time concentrating in class, while doing assignments, or while working due to smartphone use	661 (24.7)	1,065 (39.7)	410 (15.3)	222 (8.3)	265 (9.9)	59 (2.2)
Feeling pain in the wrists or at the back of the neck while using a smartphone	482 (18.0)	781 (29.1)	342 (12.8)	529 (19.7)	438 (16.3)	110 (4.1)
Won’t be able to stand not having a smartphone	525 (19.6)	784 (29.2)	407 (15.2)	268 (10.0)	472 (17.6)	226 (8.4)
Feeling impatient and fretful when I am not holding my smartphone	689 (25.7)	965 (36.0)	309 (11.5)	300 (11.2)	300 (11.2)	119 (4.4)
Having my smartphone in my mind even when I am not using it	639 (23.8)	1076 (40.1)	312 (11.6)	267 (10.0)	316 (11.8)	72 (2.7)
I will never give up using my smartphone even when my daily life is already greatly affected by it	717 (26.7)	1196 (44.6)	308 (11.5)	218 (8.1)	201 (7.5)	42 (1.6)
Constantly checking my smartphone so as not to miss conversations between other people on Twitter or Facebook	883 (32.9)	906 (33.8)	270 (10.1)	254 (9.5)	285 (10.6)	84 (3.1)
Using my smartphone longer than I had intended	346 (12.9)	837 (31.2)	328 (12.2)	500 (18.6)	557 (20.8)	114 (4.3)
The people around me tell me that I use my smartphone too much	479 (17.9)	744 (27.7)	336 (12.5)	372 (13.9)	523 (19.5)	228 (8.5)

**Table 2:** The mean of smartphone addiction score and proportion of potentially addicted students by sex, age groups and residence place.

Independent Variables		Number	Mean score (95% CI)	*P* value†	Effect size (95% CI)	Proportion of potential addiction (95% CI)	*P* value‡
Total participants		2682	27.1 (26.6–27.6)	-	-	29.8 (28.1–31.5)	-
Sex	Male	1485	28.0 (27.3–28.6)	< 0.001	0.22 (0.14–0.29) ¶	34.9 (31.5–38.4)	< 0.001
	Female	1197	26.1 (25.3–26.8)			22.3 (19.3–25.5)	
Age group(years)	10	244	25.6 (24.6–26.9)	< 0.001	0.03 (0.02–0.04) §	21.8 (15.7–29.5)	< 0.001
	11	310	24.7 (23.7–25.8)			19.6 (14.6–25.9)	
	12	407	25.8 (24.9–26.7)			21.8 (17.9–26.3)	
	13	576	24.9 (26.1–27.7)			30.0 (26.2–34.1)	
	14	597	28.1 (27.3–29.0)			32.9 (28.0–38.3)	
	15	548	29.5 (28.3–30.7)			40.2 (33.9–46.8)	
Residence Place	Urban	2367	27.1 (26.5–27.6)	0.031	0.13 (0.01–0.25) ¶	28.9 (26.3–31.5)	0.004
	Rural	315	28.2 (27.3–29.1)			36.8 (31.3–42.7)	

† Measured by T-test/ANOVA; ‡ Measured by chi square test; ¶ Cohen’s d; § Eta-squared.

**Table 3:** Pairwise comparison of mean smartphone addiction score by different age groups (years) using Scheffe method. P-values presented in parentheses.

Row Mean - Column Mean	9	10	11	12	13	14
10	-2.85 (0.878)					
11	-3.72 (0.650)	-0.87 (0.965)				
12	-2.66 (0.901)	0.18 (1.000)	1.05 (0.849)			
13	-1.54 (0.993)	1.30 (0.681)	2.18 (0.043)	1.12 (0.664)		
14	-0.34 (1.000)	2.51 (0.022)	3.38 (< 0.001)	2.32 (0.007)	1.20 (0.449)	
15	1.03 (0.999)	3.88 (< 0.001)	4.75 (< 0.001)	3.70 (< 0.001)	2.58 (< 0.001)	1.37 (0.291)

The overall prevalence of potential smartphone addiction was 29.8% (95% CI: 28.1–31.5), which was higher among boys and older students (Table 2). The prevalence of potential smartphone addiction was higher in rural (36.8%) than urban (28.9%) areas, however this difference was not significant after adjustment with other variables (*P* = 0.089). Male sex and older age were positively associated with potential smartphone addiction in multiple logistic regression model (Table 4).

**Table 4:** The association of sex, age and residence place on potential smartphone addiction in multiple logistic regression model.

Predictors		Odds ratio (95% confidence interval)	*P* Value
Sex	Female	Reference	-
	Male	1.85 (1.49–2.99)	< 0.001
Age group (years)	10	Reference	-
	11	0.90 (0.56–1.44)	0.662
	12	1.01 (0.63–1.61)	0.965
	13	1.42 (0.93–2.17)	0.101
	14	1.71 (1.08–2.72)	0.022
	15	2.40 (1.50–3.75)	< 0.001
Residence place	Urban	Reference	-
	Rural	1.26 (0.96–1.65)	0.089

The odds of potential smartphone addiction was higher in boys (OR = 1.85, *P* < 0.001). Additionally, potential smartphone addiction was higher at 14 (OR = 1.71, *P* < 0.022) and 15 (OR = 2.37, *P* < 0.000) years old students compared to the students in the 10-year group.

## Discussion

Problematic use of smartphones is increasing worldwide, and meanwhile, students are being exposed to the negative physical, behavioral, and social effects of this technology. This study revealed that approximately 30% of the students in an Iranian sample, were potentially addicted to smartphones according to their self-reports to the SAS-sv questionnaire.

The mean score of self-reported SAS-sv in this localized study in Iran was 27.12 ± 8.72 which is almost the same among university students in Turkey^10^, India^30^, and Japan^31^. However, the mean score of SAS-sv was 38.7 in a sample of university students in Jordan in 2022^32^. The most comparable studies to our results were conducted by Mokhtarinia et al.^33,34^ during 2021–2022. They reported a mean score of 32.7 and 33.7 for the SAS-sv in non-random samples of Iranian individuals aged 15–24 and 6–19 years old with no significant differences in age and sex groups. Higher mean of SAS-sv in Mokhtarinia’s studies can be attributed to their study time, which was after the COVID-19 epidemic when people used more smartphones for work or education. Other causes for discrepancies between studies may include differences in age groups, study time, target population, sampling method, and behavioral factors in smartphone usage.

In a review article in this field, the prevalence of problematic use of smartphones in Iran was between 0.9% and 64.5%^35^. The prevalence rate of smartphone addiction was reported as 19.9% in Switzerland^36^, 12.5% in Spain, and 21.5% in Belgium^37^. The wide range in the prevalence of smartphone addiction may be due to differences in the study population and sampling method, the measurement tools, Internet access, and the study year. According to studies conducted using the SAS-sv questionnaire, the prevalence rate among young people was 29.8% in China^38^, 33.1% in Brazil^39^, 39.8% in Turkey^40^, 44.6% in Lebanon^41^, and 46.9% in Malaysia^42^. The prevalence of smartphone addiction were 48.3% and 53.3% in recent studies with similar cut-points in SAS-sv among Iranians aged 15–24^33^ and 6–19^34^ years old. These rates are higher than our estimate and confirm the hypothesis that problematic use of smartphones has increased after COVID-19 pandemic^34,43^.

In this study, it was found that the odds of potential smartphone addiction in boys was significantly higher than in girls, consistent with the results of other studies^30,44^. However, another study, reported that girls are more likely to be addicted than boys^31,45^. Some studies also reported that gender plays no role in the prevalence of smartphone addiction^33,34,41,46^. One possible explanation for these discrepancies could be variations in the age of participants across different studies. Factors such as greater tendency for girls to use social networks^47^, the existence of personality traits like neuroticism and stress^48^, and increased time spent at home (leading to virtual communication replacing face-to-face communication with friends) may contribute to higher prevalence of the smartphone addiction in older girls. Conversely among younger students, (like those in our study), certain personality traits may not have fully emerged, families may exert more control over their daughters’ smartphone use, and cultural differences in societies could also play a role., Additionally boy’s interest in playing digital games on smartphones from a young age^49^ might contribute to the lower prevalence of smartphone addiction among female students in the present study. Furthermore, the choice of a higher cutoff for girls in this study could be a reason for the higher prevalence of smartphone addiction in boys. Therefore, it may be necessary to reconsider the selection of the appropriate cutoff for identifying potentially addicted individuals across both genders, taking into account the specific conditions of each society.

In this study, where the participants were in the age range of 10 to 15 years, it was found that the addiction prevalence increased between the ages of 14 and 15 compared to the age of 10 year. This rise may be attributed to allowing parents to give their children more access to smartphones as they get older, the appeal of phone use and gaming, improved students proficiency in utilizing phone features, and increased exposure to new technologies. Additionally, internet and social networks usage tends to rise with age. The association of age and phone addiction has been investigated in other studies, some studies have indicated that the prevalence of phone addiction tends to increase with age^39,50,51^. However, some studies indicate that smartphone addiction dose not significantly associate with the age^10,30,33,52^. A similar study in Iran showed that the prevalence of smartphone addiction decreased from 63.2% among 6–11 years old, to 53.6% among 12–14 years old, and further to 51.4% among 15–19 years old children^34^. It has been shown that the association of age and smartphone addiction is different in different periods of life. During childhood and adolescence, the phone addiction tends to increase with age. However in adulthood, age does not appear to have any association with phone addiction^52,53^. Given the age range of the students in this study, the higher odds of addiction among older students seems reasonable.

After adjusting for age and sex of the participants, the place of residence didn`t show a significant association with phone addiction in the present study. However, another study reported that the smartphone addiction was higher in urban residents than in rural areas^54^. This discrepancy may be attributed to differences in internet access, available alternatives for information and communication, and leisure opportunities between rural and urban populations. As a result, the prevalence of smartphone addiction in urban and rural regions may vary.

This study investigated the association between place of residence, age, and sex groups with potential smartphone addiction. Based on these findings, new interventions can be developed by considering more vulnerable groups. However, it is important to note that the impact (as measured by effect sizes) of studied variables is relatively small. To create more effective interventions, it is essential to investigate other influencing factors. One of the strengths of this study is the investigation of potential smartphone addiction in a population-based study with a sufficient sample size.

By comprehensively analyzing the latest state of smartphone use among students and examining its associated factors, as well as calculating the effect sizes of the covariates, this study can provide valuable insights for designing appropriate interventions to control smartphone addiction. Additionally, using a valid and reliable questionnaire and completing it carefully with a good supervision is another strength point of this study. Limited age range of participants and the lack of recording data for daily use of the internet, mobile phones and other related technologies such as computers and tablet were the main limitation of present study. Furthermore, we did not measure the spending time for social networks, doing homework or using entertainment and games, as well as physiological and social behavior and performance of students, which can be considered as another limitations. Finally, it is important to note that the results of this cross-sectional study cannot be interpreted as indicating a causal relationship between variables. Therefore, any attempt to draw a causal inference should be avoided.

## Conclusion

About one third of 10 to 15 years old Iranian students have potential addiction to smartphones in 2018. Potential addiction was more common in boys compared to girls and in 14 to 15 year olds compared to younger students. It is recommended to develop appropriate interventions for the use of this growing and evolving tool, as well as monitoring the state of addiction in the coming years.

## Electronic supplementary material

Below is the link to the electronic supplementary material.

Supplementary Material 1

## Data Availability

The datasets analyzed during the current study are available from the corresponding author on reasonable request.
