# Supplementary material for: Smartphone addiction in Iranian schoolchildren: a population-based study
Source: Sci Rep. 2024 Sep 27;14:22304. doi: 10.1038/s41598-024-73816-8 (PMC11436884; doi:10.1038/s41598-024-73816-8)
Supplement: Supplementary file 1 — Supplementary Material 1 [file 41598_2024_73816_MOESM1_ESM.docx]

| **Appendix 1. English Version of SAS-SV** | | | | | | | |
| --- | --- | --- | --- | --- | --- | --- | --- |
| **Items** | | **Strongly disagree** | **Disagree** | **Weakly disagree** | **Weakly agree** | **Agree** | **Strongly agree** |
| 1 | Missing planned work due to smartphone use | 1 | 2 | 3 | 4 | 5 | 6 |
| 2 | Having a hard time concentrating in class, while doing assignments, or while working due to smartphone use | 1 | 2 | 3 | 4 | 5 | 6 |
| 3 | Feeling pain in the wrists or at the back of the neck while using a smartphone | 1 | 2 | 3 | 4 | 5 | 6 |
| 4 | Won’t be able to stand not having a smartphone | 1 | 2 | 3 | 4 | 5 | 6 |
| 5 | Feeling impatient and fretful when I am not holding my smartphone | 1 | 2 | 3 | 4 | 5 | 6 |
| 6 | Having my smartphone in my mind even when I am not using it | 1 | 2 | 3 | 4 | 5 | 6 |
| 7 | I will never give up using my smartphone even when my daily life is already greatly affected by it. | 1 | 2 | 3 | 4 | 5 | 6 |
| 8 | Constantly checking my smartphone so as not to miss conversations between other people on Twitter or Facebook | 1 | 2 | 3 | 4 | 5 | 6 |
| 9 | Using my smartphone longer than I had intended | 1 | 2 | 3 | 4 | 5 | 6 |
| 10 | The people around me tell me that I use my smartphone too much. | 1 | 2 | 3 | 4 | 5 | 6 |
